# Stability of the Soft Sparking State in the Plasma Electrolytic Oxidation Process

**DOI:** 10.3390/ma18050989

**Published:** 2025-02-24

**Authors:** Stanisław Pietrzyk, Wojciech Gębarowski

**Affiliations:** 1 Department of Physical Chemistry and Metallurgy of Non-Ferrous Metals, Faculty of Non-Ferrous Metals, AGH University of Krakow, al. Adama Mickiewicza 30, 30-059 Cracow, Poland; 2 Independent Researcher, V-Lab, 30-437 Cracow, Poland

**Keywords:** plasma electrolytic oxidation, aluminum, alumina coatings, soft-sparking phenomenon, cathodic current

## Abstract

Soft sparking is a phenomenon observed during plasma electrolytic oxidation (PEO) performed under alternating current (AC) conditions. It is directly associated with the presence of cathodic polarization during the oxidation process, contributing to enhanced functional coating properties. However, the role of cathodic current in oxide-layer formation remains ambiguous. This study presents findings suggesting that soft sparking is a dynamic equilibrium state occurring within a certain stability window, primarily governed by the anodic to cathodic charge ratio in AC cycles. By analyzing soft-sparking behavior under varying cathodic-to-anodic charge ratios, frequency, cathodic pulse duty cycles, and alkalinity of electrolytes, the proposal of a mechanism underlying this process is presented. The authors suggest that the soft-sparking state may be linked to the formation of active sites during cathodic polarization and their subsequent suppression during anodic polarization. This occurs due to oxidation and deposition of Al(OH)_3_, facilitated by localized OH^−^ ion accumulation in these regions. Additionally, the restricted diffusion of water molecules toward the substrate may play a crucial role in sustaining the soft-sparking state.

## 1. Introduction

Plasma electrolytic oxidation (PEO) is a method for producing oxide coatings on passivating metal substrates through electrochemical oxidation in aqueous electrolytes. This process occurs at anodic voltages exceeding the breakdown voltage of the oxide layer forming on the electrode surface. Such oxidation conditions result in a complex mechanism that involves not only electrochemical reactions but also physicochemical processes related to the presence of high temperature generated by plasma micro-discharges in the oxide layer. Oxide coatings produced through the PEO process exhibit properties that make them suitable for various applications, particularly as wear-resistant, corrosion-resistant, thermal-resistant, and electrical insulating coatings. The PEO method has been widely discussed in the literature and comprehensively reviewed by Yerokhin et al. [1], Jiang and Wang [2], Simchen et al. [3], Fernández-López et al. [4], and Mojsilović et al. [5]. The PEO process can be performed under different electrical conditions, which may be divided into two groups: direct current (DC) and alternating current (AC). The DC mode encompasses pulsed current techniques and various current modulation methods that do not alter the direction of the electric current flow. In contrast, the AC conditions include all electrical modes that incorporating cathodic polarization pulses, regardless of the polarization waveform shape used. In general, AC PEO allows thicker layers to be produced with higher micro-hardness [6], lower porosity [7], improved thickness uniformity [8], stronger adhesion to the substrate [9], and enhanced wear and corrosion resistance [10,11,12]. Therefore, AC PEO is considered more effective for obtaining high-quality protective coatings. The role of polarization waveforms on PEO was recently reviewed by Dogadkin et al. [13]. Since cathodic polarization significantly influences the oxide-layer formation mechanism, investigating the role of cathodic current in the PEO process is essential.

During AC PEO, a phenomenon named “soft sparking” may occur. Although an unequivocal definition has not yet been proposed, it is generally associated with the occurrence of a new type of micro-discharges during oxidation. These micro-discharges are characterized by a continuous spectrum of light emission and will be referred to as “soft type” micro-discharges. Different aspects of soft sparking were described by several authors [7,13,14,15]. The phenomenon was reported mainly for aluminum alloy substrates, but also for magnesium alloys [16]. When AC PEO is carried out under specific electrical conditions, i.e., the cathodic current density exceeds the anodic one, a rapid decrease in anodic breakdown voltage is observed at a certain stage of layer formation. This decrease in anodic voltage is accompanied by the disappearance of the regular type of micro-discharges and transition to only soft-type micro-discharges. The name “soft sparking” originates most likely from the fact that these micro-discharges generate much lower acoustic emissions comparing to the regular-type ones [7]. It should be underlined that the soft-sparking type of micro-discharges may occur (along with other type of micro-discharges) also before this transition, which would be useful to denote as a “soft-sparking state” (abbreviated as SSS in this paper) when no other type of discharges occurs simultaneously. Hence, even without distinct transition to SSS, the coating microstructure, composition, and thus its properties may be partly affected by soft sparking. Therefore, it would be appropriate to distinguish two states: the full soft-sparking state and partial soft sparking, when two different types of micro-discharges coexist. The role of cathodic current is crucial for understanding the soft-sparking phenomenon, and it has recently been summarized by Rogov et al. [17,18], who, based on their research and hitherto works, proposed a phenomenological concept of mechanisms during soft sparking [17]. They suggested that soft sparking may be described by potential barriers in specific regions at the metal–oxide and oxide–electrolyte interfaces. According to Rogov et al., the cathodic polarization causes local acidification with respect to the aluminum oxide isoelectric point (estimated at pH ≈ 9.1 [19]), which diminishes the potential barrier in the electrical double layer. The authors used the terms “active zone” and “product zone” to describe the inner dense part and the outer porous part of the oxide layer, respectively. The active zone shifts with the front of the oxide-layer growth inward in the substrate. According to the presented mechanism, at a certain coating thickness, a proton injection during the cathodic polarization can produce relatively stable [H•]_OX_ complexes in the active zone. This results in the formation of regions enriched with [H•]_OX_ complexes, with increased conductivity, and it is responsible for the observed drop in anodic voltage after the transition to SSS. In subsequent anodic polarization, the [H•]_OX_ dissociation and outward proton migration take place, which suppress the breakdown processes. The proposed mechanism seems to be incomplete, as the alternating reduction in protons to [H•]_OX_ complexes and their reverse oxidation to protons during cathodic and anodic polarization, respectively, do not explain the fact that the growth rate of coatings increases with increasing current of cathodic polarization [10,20]. Therefore, additional processes during soft sparking, involving oxygen-bearing species that are responsible for facilitating the oxide layer growth, must be taken into consideration.

The AC conditions during PEO can be implemented using different pulse shapes, frequencies, duty cycles, and patterns of anodic and cathodic pulses. While the wide range of electrical parameters allows for extensive optimization of the PEO process, it also complicates the interpretation of experimental data found in the literature, especially when these parameters are not described in detail. Moreover, the PEO process may be influenced by unique characteristics of the power sources used, which can respond differently to dynamically changing impedance of the electrolytic cell. This effect is difficult to quantify and introduces additional uncertainties when comparing data.

In this study, the authors present a new perspective on soft sparking and propose a conceptual model for oxide-layer formation in PEO under AC conditions, particularly during SSS.

## 2. Materials and Methods

Samples with dimensions of 3 × 1 × 0.15 cm made of aluminum 1050 A were used as electrodes, with a total working surface area of 7.13 cm^2^. Prior to oxidation, samples were prepared in the following steps: degreasing in acetone, etching in 0.25 M NaOH solution, brightening in 7 M HNO_3_ solution, rinsing in deionized water. The sample surface preparation procedure was more extensive than the standard approach for the PEO process to assure good reproducibility of tests, particularly for high-voltage cyclic voltammetry and electrical breakdown tests. Alkaline–silicate electrolytes with a composition 0.035 M KOH, 0.08 M Na_2_SiO_3_ were used as the electrolytes for all experiments except for the one where different alkalinity was used. A 4 dm^3^ stainless steel container was used as an electrolytic cell and served as the counter electrode. The electrolytes were mechanically stirred, and its temperature was maintained in the range of 23–26 °C using a water-cooling system.

PEO process was performed using a system consisting of two programmable DC laboratory power supplies (Chroma ATE, Taoyuan, Taiwan) connected to the electrolytic cell through transistor switch with a full bridge topology. Each power supply unit controlled anodic and cathodic current independently. The switch allowed for toggling between two units with different frequencies and duty cycles, generating the anodic and cathodic pulses patterns required for PEO. An FPGA-based platform (National Instruments, Austin, TX, USA) with a real-time operating system (LabVIEW 2012 with Real-Time & FPGA Module) was used for controlling the switch and for recording voltage and current during the process. The described setup generated square-like voltage pulses for both anodic and cathodic current directions. The voltages of pulses were controlled to provide constant total charge during each pulse. Therefore, within pulses the oxidation occurred at constant voltage, while considering a longer time period, constant average current densities were maintained—such conditions can be characterized as quasi-galvanostatic. The oxidation process was carried out using different electrical parameters, such as current density, frequency, cathodic-to-anodic charge ratio, and cathodic pulse duty cycle. Specific electrical parameters for each experiment are included in the captions of the figures in the next section. The cathodic-to-anodic charge ratio (R_C/A_) is defined as:

(1) $$R_{C/A}=\frac{Q_{C}}{Q_{A}}=\frac{j_{C\;av}}{j_{A\;av}}$$

where Q_C_ and Q_A_ are the total charge during cathodic and anodic cycles, respectively. It can also be expressed as the average cathodic (j_C av_)-to-average anodic (j_C av_) current density ratio. The anodic and cathodic pulse duty cycles, denoted as D_A_ and D_C_, respectively, express a percentage ratio of anodic and cathodic pulse time to the total period time. Polarization sweeps in the range of 0 to 600 V anodic voltage with a scan rate of 3 V·s^−1^ were performed in the same cell as PEO during the temporary interruption of the oxidation process.

SEM observations with EDS analysis were performed using a Hitachi SU-70 scanning electron microscope, Hitachi, Ltd., Tokyo, Japan. Samples were sputtered with gold prior to SEM observations. Phase composition was determined by X-ray diffraction using a Rigaku MiniFlex diffractometer, Rigaku Corporation, Tokyo, Japan with Cu Kα radiation. Thin layers of the oxide coating were subsequently removed mechanically in order to perform diffraction analysis of bulk oxide on different depths.

## 3. Results and Discussion

### 3.1. Stability of Soft-Sparking State

**Figure 1:**
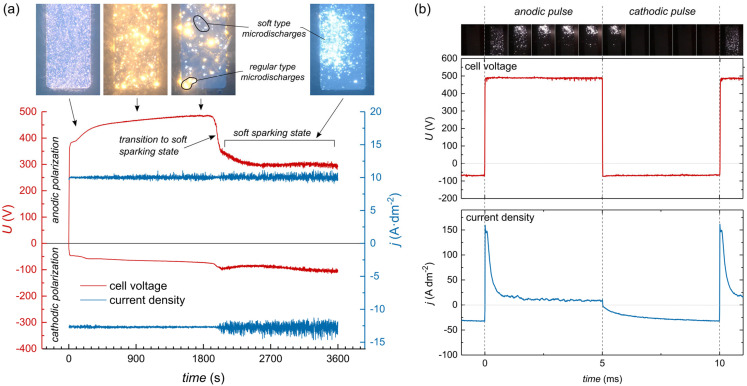
(**a**) Cell voltage and average current density of AC PEO during whole process; waveform of voltage and current density during a single cycle; (**b**) PEO process parameters: j_A av_ = 10 A dm^−2^, f = 100 Hz, R_C/A_ = 1.25, D_A_, D_C_ = 50%. Exposure time of photos: (**a**) 20 ms, (**b**) 0.5 ms.

Figure 1 presents the anodic and cathodic voltages and current densities during PEO under AC conditions with an R_C/A_ = 1.25. Under these conditions, the transition to SSS occurred around the 33rd minute of oxidation, marked by a distinct decrease in anodic voltage from approximately 480 to 300 V.

**Figure 2:**
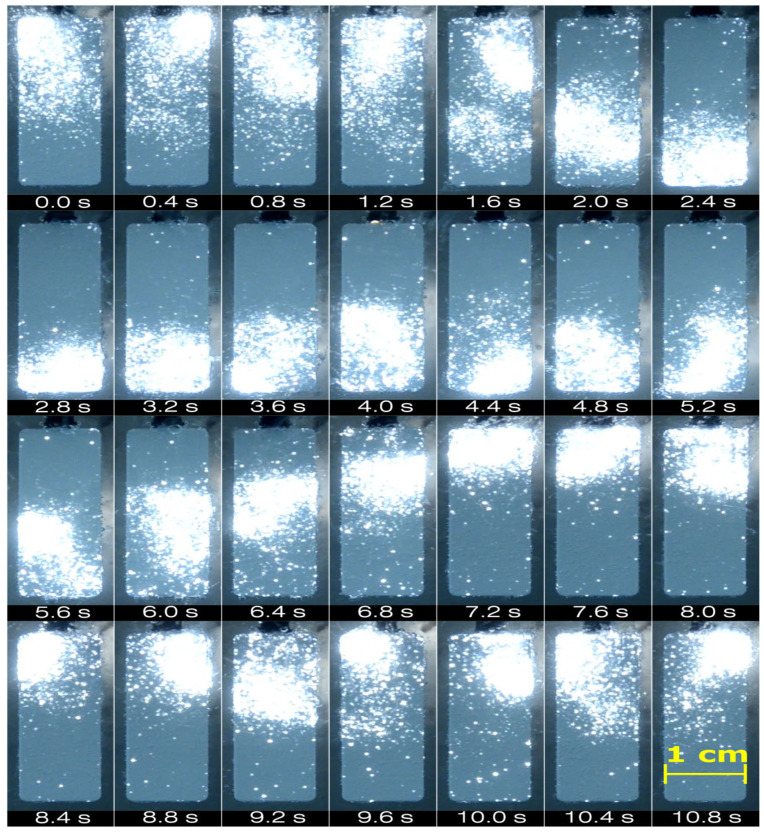
The photo sequence of micro-discharge movement during SSS. Exposure time of photos: 20 ms.

The images above the plot show micro-discharges on the electrode surface during various stages of the PEO process. Before the transition to SSS, two distinct types of micro-discharges were visible: larger yellowish (regular type) discharges and smaller whitish (soft type) discharges. As time progressed, the proportion of soft-type discharges increased, while regular discharges diminished. After transitioning to SSS, regular micro-discharges disappeared completely, and only soft-type micro-discharges remained on the electrode surface. This change in micro-discharge type, correlated with the reduction in anodic voltage, indicates that soft-type micro-discharges occurred at a much lower ohmic resistance of the oxide layer. Soft-type micro-discharges differed from the regular ones not only in appearance but also in spatial occurrence. Instead of the random occurrence of micro-discharges across the entire electrode surface, they were concentrated in a smaller area at a time, continuously sweeping the whole electrode surface. The sequence of photos in Figure 2 shows this characteristic discharge movement, which was present during the whole SSS. This phenomenon is always observed during SSS in the PEO process run in the presented conditions.

It is a recognized fact that the transition to SSS occurs only if the cathodic current or cathodic charge exceeds the anodic one. A previous author’s study [20] showed that the transition to SSS was achieved for R_C/A_ ≥ 1.25 (in the cited paper, ratio was expressed as anodic to cathodic; hence, here it is a reciprocal of R = 0.8), and the case of R_C/A_ ≤ 1.11 resulted in soft sparking, which developed only on a part of the electrode and coexisted with regular-type micro-discharges that became larger with time and finally formed arcs that locally destroyed the oxide layer. Approaching the minimal value of R_C/A_ required for SSS (value between 1.11 and 1.25), the system went into an instable region, jumping between two states, and the anodic voltage oscillated between normal-state anodic voltage (~480 V) and the SSS value (~300 V). In order to estimate more precisely the limit value of R_C/A_ at which SSS can still be maintained, the following procedure was performed. In the first step, a stable SSS was achieved using R_C/A_ = 1.25, and then the cathodic current density was gradually decreased (R_C/A_ was decreased) until a stable SSS was lost, which was associated with the reappearance of regular-type micro-discharges, an increase in anodic voltage, and an increase in acoustic emission. This value of R_C/A_ was considered the minimal cathodic-to-anodic charge ratio required for sustaining SSS. The above procedure was repeated for different anodic current densities. Experiments revealed that the minimal value of R_C/A_ was around 1.06 and almost did not depend on the anodic current density in the tested range of j_A av_ = 2.5–11.5 A dm^−2^. The obtained value of R_C/A_ shows that SSS requires a higher value of R_C/A_ for the activation than later for the maintenance, and it has a certain stable region that is defined by the ratio of charge in anodic and cathodic pulses. This indicates that the stability of SSS may be balanced by the amount of substrate/products of reactions occurring during the anodic and cathodic cycles.

**Figure 3:**
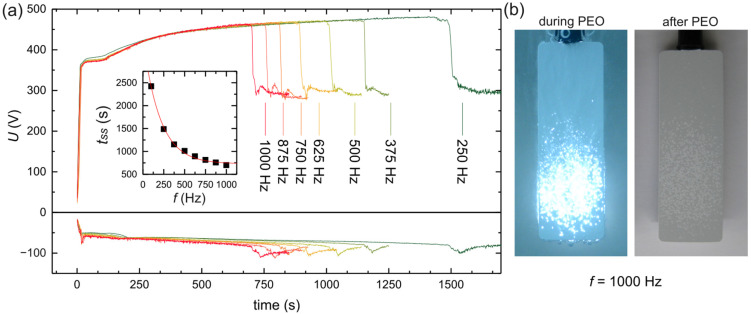
The effect of increasing frequency on the time of soft-sparking transition: (**a**) cell voltage during PEO and time to transition (tss) plotted vs. frequency; (**b**) photos of the electrode oxidized at f = 1000 Hz during and after the process. PEO process parameters: j_A av_ = 10 A dm^−2^, R_C/A_ = 1.25, DA, DC = 50%. Exposure time of photos: 20 ms.

**Figure 4:**
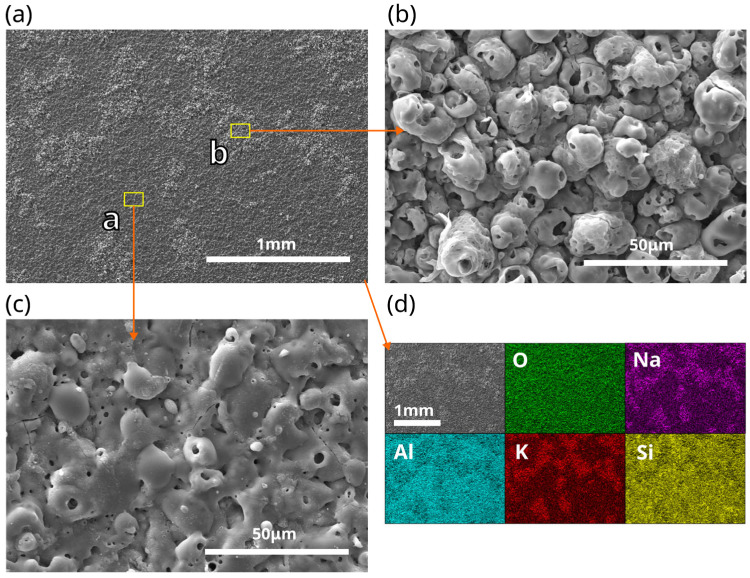
SEM images with EDS mapping of the electrode surface shown in Figure 3b. (**a**) whole analyzed section; (**b**) region “b” in magnification, covered by micro-discharges during SSS; (**c**) region “a” in magnification, not covered by micro-discharges during SSS; (**d**) EDS surface mapping of the whole section.

When higher frequencies were applied during PEO (keeping the rest of the electrical parameters unchanged), the time to the transition to SSS became shorter. Figure 3a shows the voltage curves for different AC frequencies during the process time until SSS was achieved. The time to the transition (tss) decreased exponentially with increasing frequency. However, at higher frequencies, micro-discharges during SSS were sweeping not the whole but only a part of the electrode area, and the rest of electrode stayed inactive. The higher the AC frequency, the larger the inactive area during SSS. The area where soft-type micro-discharges were sweeping could easily be distinguished as the lighter white spots on the electrode surface after the process. The photos in Figure 3b show a sample oxidized at a frequency of 1000 Hz during SSS and after oxidation. The SEM images of the electrode surface (Figure 4) reveal these lighter spots to be more porous regions with a rougher structure that consists of nodular formations with discharge channels in them (region “b” in Figure 4), in comparison to a region not covered by discharges during SSS (region “a” in Figure 4). Active regions were elevated in relation to the level of inactive regions on the electrode, indicating a higher growth rate of oxide in these places. The question is whether the structure shown as region “b” was freshly formed during SSS or came from a conversion of the structure shown as region “a”, which underwent cracking, uplifting, and chemical dissolution by the electrolytes. Considering that the oxide film grew an inward substrate and the soft type of micro-discharges during SSS were smaller and occurred in deeper regions of the oxide layer, the structure of outer layer should not have been affected directly by them. Additionally, if the oxide material surrounding the discharge channels was less hydrated due to the presence of high temperature in these regions, more hydrated oxide material could be dissolved preferentially, leaving nodular formations that, in fact, are old discharge channels along with the outlining material.

The SEM-EDS mapping of the electrode surface, shown in Figure 4, indicates a higher concentration of sodium and potassium in the brighter areas. Since these cations are the main positive charge carriers present in the electrolytes, they are being attracted to the active regions where the reactions occur exclusively.

The whole surfaces of the samples oxidized at lower AC frequencies (e.g., at f = 100 Hz, without the anomaly described above) look like region “b” from Figure 4. This shows that soft sparking develops in highly porous structures, forming them simultaneously. Considering that this porous structure had higher volumetric capacity, SSS could be related to the electrode capacity, and therefore, it is not a certain thickness, as previously suspected, but rather a certain volume of oxide layer that is required for maintaining SSS. The higher the switching frequency, the smaller the oxide-layer capacity (area of electrode) needed for maintaining SSS; hence, the aspect of mass transport through layers and the accumulation of reaction products within layers may play key roles in the mechanism of soft sparking.

### 3.2. Morphology of Oxide Layers

**Figure 5:**
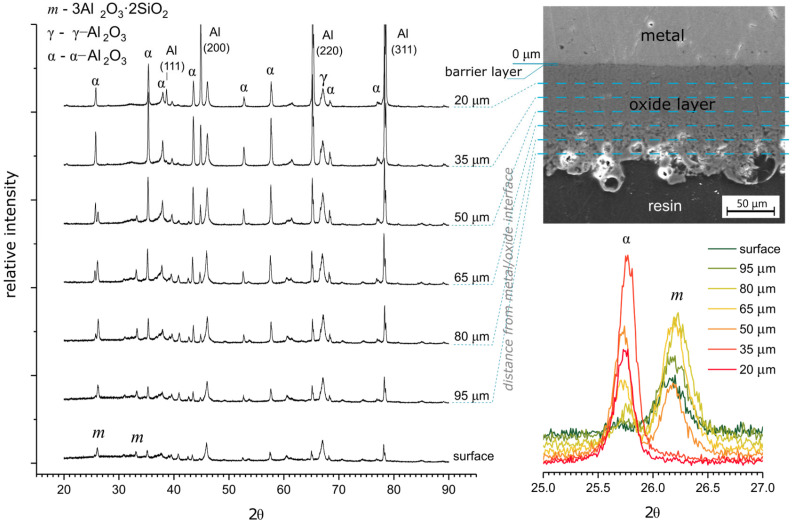
XRD analysis of the oxide layer at different depths from the top surface into the bulk. PEO process parameters: j_A_ = 10 A cm^−2^, f = 100 Hz, R_C/A_ = 1.25, D_A_, D_C_ = 50%, t = 60 min.

In general, the very porous structure of the oxide layer surface after soft sparking, described in the previous paragraph, constitutes only the topmost layer of the total coating thickness, which has poor mechanical properties. Beneath this porous layer, the oxide exhibits a more solid structure. Figure 5 presents the phase analysis of the bulk oxide of the formed layer at different depths. On the surface and in the outer parts of the layer a mullite and γ-Al_2_O_3_ phases are predominant. However, going toward substrate, the mullite phase contribution decreases at the expense of the increasing amount of α-Al_2_O_3_ phase. In deep parts of the layer, below 35 μm from the oxide/metal interface, the mullite phase is absent and the layer is composed of only α- and γ-Al_2_O_3_. Diffractograms also show that the closer to the surface the more amorphous the layer is while going into the bulk, the higher the number of crystalline phases. In the case of oxide layers produced in DC or the pulsed mode regime (without the presence of cathodic current and with the rest of process parameters kept unchanged), they do not exhibit such a composition gradient. They have constant composition over the cross-section of the layer with a predominant mullite phase. This difference in silicon content gives a clear indication that different anionic species are involved in anodic reactions during layer formation under AC and DC conditions.

### 3.3. Processes During Anodic and Cathodic Polarization

**Figure 6:**
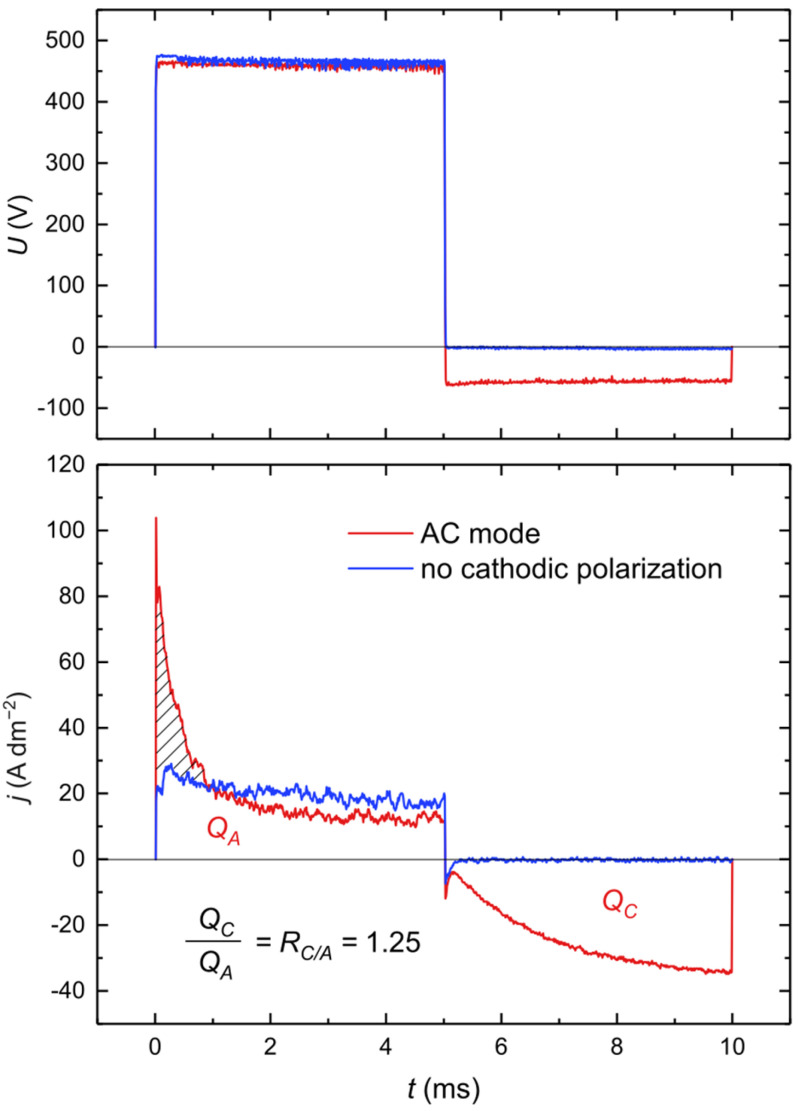
Cell voltage (**top**) and current density (**bottom**) during one cycle from the 10th minute of PEO with and without the presence of cathodic polarization. Process parameters: j_A_ = 10 A dm^−2^, R_C/A_ = 1.25, f = 100 Hz, DA, DC = 50%.

Figure 6 presents the cell voltage (top) and the current density (bottom) waveforms during a single cycle from the 10th minute of AC PEO (red curve) and with the cathodic polarization intermittently turned off (blue curve). Since the utilized setup maintains constant voltage during pulses, almost ideal square waveform of the cell voltage is observed on the top plot. Considering that the voltage is constant within a pulse duration, the current response directly represents instantaneous cell impedance changes. The areas under the current curve denoted as Q_A_ and Q_C_ represent the total charge during anodic and cathodic pulses, respectively. When the cathodic polarization (pulse) is absent, the anodic current during the anodic pulse is relatively flat, with a little noise from micro-discharge events. However, when the cathodic polarization is present, at the beginning of the anodic pulse, a large current spike is formed. This shows that the cathodic pulse causes a high current flow at the beginning of the anodic pulse. This may be related to (i) recharging of the electrode capacitance, which was charged with reverse polarity in the previous cathodic pulse, or (ii) fast oxidation of products from reactions during the previous cathodic pulse and accumulated at the interface. The effective electrode capacitance is considered oxide layer capacitance and double-layer capacitance connected in series. The charge corresponding to the peak, marked as the dashed area on the plot in Figure 6, is about 1.85 × 10^−4^ Q cm^−2^. Since the average voltage is 462 V, the capacitance related to this charge can be calculated from formula C = Q/V ≈ 400 nF cm^−2^. The electrode capacitance at this stage of PEO estimated by electrochemical impedance spectroscopy (EIS) measurements performed in stationary conditions in 0.1 M Na_2_SO_4_ is about 90 nF cm^−2^ [21]. Recent measurements of the electrode capacitance by the authors (not published) performed in the same electrolytes used during oxidation and with a shorter interval time between PEO and EIS showed a slightly higher value—about 100 nF cm^−2^, but this value can still be underestimated, considering that electrode capacitance during PEO can be higher due to adsorption processes during cathodic polarization. However, assuming similar values of the electrode capacitance during the oxidation process, the current peak at the beginning of the anodic cycle is composed of both charging and Faradaic current.

The shape of the current curve shows a different kinetic trend of reactions during the cathodic pulse comparing to the anodic one. Except for a slight peak at the beginning, the current increases continuously throughout the course of the cathodic pulse. Since cations such as Na^+^ and K^+^ cannot be reduced and the concentration of protons is relatively low (electrolyte pH ≈ 12), the main reaction that occurs during the cathodic pulse is the electrochemical reduction of water molecules to gaseous hydrogen and hydroxide anions based on the overall hydrogen evolution reaction:

H_2_O + e^−^ ⇄ 0.5H_2_ + OH^−^, (2)

**Figure 7:**
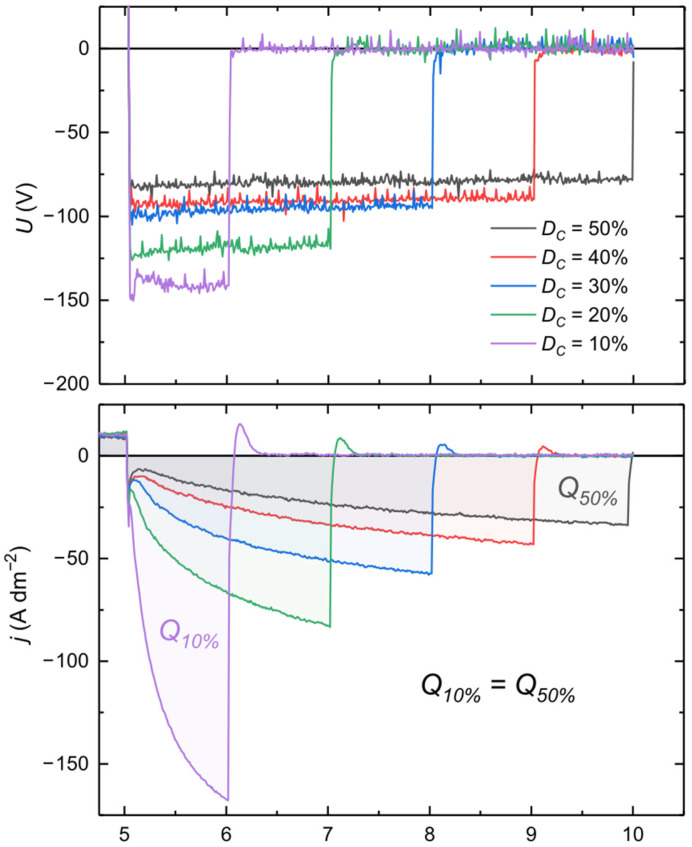
Cell voltage (**top**) and current density (**bottom**) during cathodic pulses with different cathodic pulse duty cycles (DCs) during SSS. Process parameters: j_A av_ = 10 A dm^−2^, f = 100 Hz, R_C/A_ = 1.25, D_A_ = 50%.

The increasing cathodic reaction rate with time may be explained as follows. At the beginning of the cathodic pulse, the barrier layer is tight and the diffusion of water to the metal/oxide interface is limited; thus, the current is low. The current starts to flow through weaker spots of the oxide (defects in the structure), and first-order water molecules that are the closest to the interface are reduced. Since the barrier layer consists of a large part of hydrated alumina, those water molecules come from Al(OH)_3_ and should be denoted as [H_2_O]_OX_. Reduced hydrogen induces mechanical stress, creating cracks and pits within the barrier layer. These defects facilitate the diffusion of water to the interface in these active spots, increasing the reaction rate with time. A similar mechanism was described by Takahashi et al. [22]. Since cathodic polarization leads to the formation of active sites in the oxide layer, which should have lower ohmic resistance, it is reasonable to conclude that in these spots the anodic reactions take place preferentially in the subsequent anodic pulse. In turn, Sah et al. [23] suggested that during a cathodic pulse, the formation of nanoporous oxide film at the bottom of the discharge channels, caused by micro-discharges in the previous anodic pulse, takes place. This nanoporous film becomes more resistive to anodic breakdowns than other places; hence, cathodic polarization during AC PEO is responsible for the randomization of spots for subsequent micro-discharges. To verify whether micro-discharges occur in active cathodic breakdown places according to Sah et al.’s theory, an experiment with different DC applied during SSS was performed. The graph depicted in Figure 7 shows the voltage and current waveforms during a cathodic pulse with different durations of 5, 4, 3, 2, and 1 ms, which correspond to 50, 40, 30, 20, and 10% D_C_, respectively, at f = 100 Hz. Q_xx%_ represents the total charge of the cathodic pulses.

**Figure 8:**
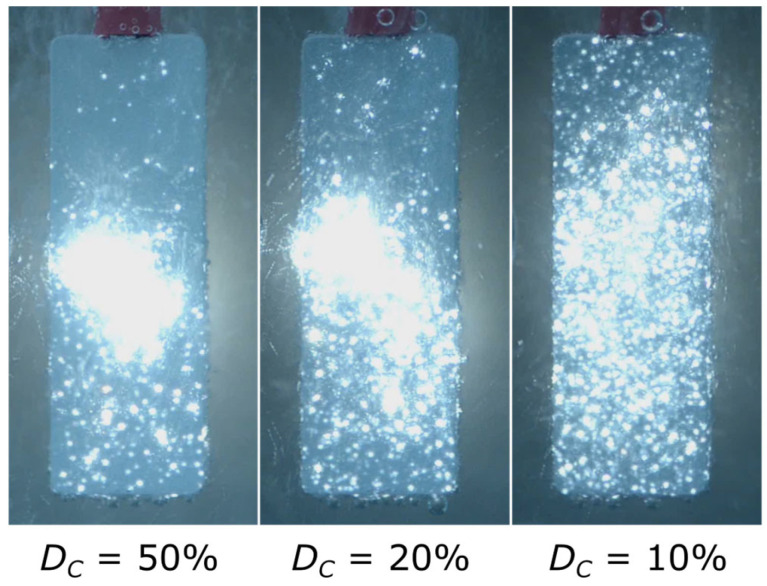
Effect of the cathodic pulse duty cycle on the dispersion of micro-discharges during SSS with different cathodic duty cycles (DCs). Exposure time of photos: 20 ms.

Since j_C av_ was the same, the total charge in the pulse (Q_xx%_) was equal for all cases. To maintain the same charge during cathodic pulses with different durations, higher instantaneous current for shorter pulse duration is required. A higher cathodic current forces the creation of a larger number of active sites on the electrode where a hydrogen evolution reaction can take place. The photos in Figure 8 show the spatial distribution of micro-discharges on the electrode surface for different DCs. It is clearly visible that at lower pulse duty cycles, the arrangement of micro-discharges became more dispersed. Therefore, it can be concluded that the active sites created during a cathodic pulse become the places where micro-discharges are initiated in the following anodic pulse. The question arising is why micro-discharges change place since active sites should also be more “attractive” for a cathodic reaction.

**Figure 9:**
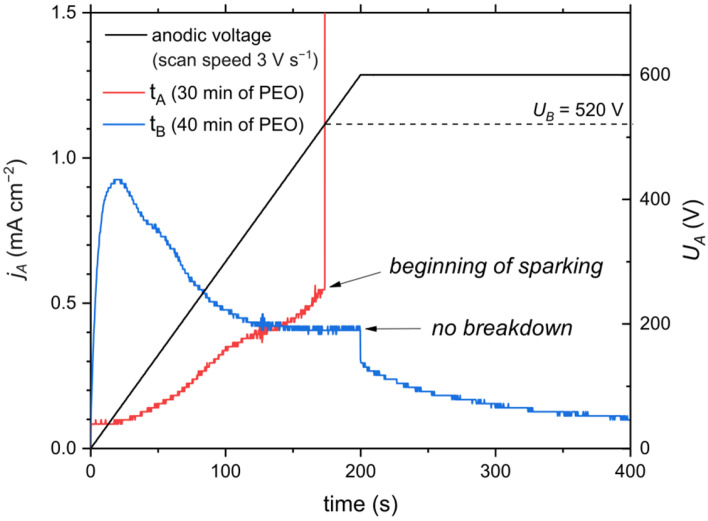
Linear anodic polarization scans of electrodes with an oxide layer produced in AC PEO for 30 min (before SSS) and 40 min (after transition to SSS).

In Figure 9, the linear scans of anodic polarization in the range of 0–600 V of samples from two different stages of PEO (marked also in Figure 1 as t_A_ and t_B_) are presented. Sample “A” came from the 30th minute of PEO—thus, before SSS transition, whereas sample “B” came from the 40th minute of PEO—after SSS transition. In the case of sample “A”, the current density increased gradually with the increase in anodic voltage until the breakdown voltage of oxide film was reached; then, a sharp increase in current was observed. The film was broken down at 520 V (some single sparks appeared slightly earlier, causing a tiny current peak, but the oxide film recovered), which is a slightly higher value than the anodic voltage at this stage of PEO (~480 V). In case of sample “B”, at the beginning of anodic polarization, the current density increased rapidly, reaching almost 1 mA cm^−2^, and then declined asymptotically. Interestingly, the oxide layer did not undergo electrical breakdown within the scan range, despite the anodic voltage being approximately 300 V at this stage of PEO. The oxide layer had initially lower resistance due to active sites in the barrier layer that were sealed by the formation of fresh hydroxide/oxide after applying anodic polarization. Therefore, anodic polarization has a “sealing effect” via the oxidation of active sites that are created during cathodic polarization. Since sealed active sites have very high resistance, indicated by the lack of breakdown up to 600 V, such an unsealing–sealing mechanism is responsible for the randomization of micro-discharges. It is worth noting that active sites do not necessarily need to change spots of occurrence in each cycle of AC during PEO. Depending on the size of the active site, this complete process, from creation to suppression of the active site, may occur over more AC periods. It was reported previously by Nominé et al. [24] that micro-discharges form localized discharge cascades consisting of up to hundreds of individual discharges and can persist at the same location over several AC voltage cycles. For the present work, a similar observation was made for SSS based on micro-discharge image analysis, where the lifespan of the active site was up to several hundreds of milliseconds.

The oxidation processes in active spots are facilitated by higher concentration of OH^−^ ions in these places as a product of the cathodic reaction (2) and may occur according to the following overall anodic reaction:

Al + 3OH^−^ ⇄ Al(OH)_3_ + 3e^−^, (3)

Hydroxide anions constitute a perfect electron donor for anodic reactions due to their very high mobility and oxygen content; hence, a sharp anodic current peak at the beginning of an anodic pulse (visible in Figure 6) may be partially caused by rapid oxidation of OH^−^ located in the vicinity of the interface. Since silicon was not detected in deeper parts of the coating (Figure 5), electrolyte anions containing silicon (e.g., [SiO_2_(OH)_2_]^2−^) did not take part in the anodic reactions in the AC PEO, except for the early stages of the process. Due to the local character of the oxidation process and consequently large local current density, the formation of micro-discharges in these spots occurred. In the course of an anodic pulse, the OH^−^ ions accumulated in the vicinity of the barrier layer are consumed by the anodic reaction (3). During SSS, when the cathodic current is being deliberately disabled intermittently, there is no production of fresh anions in the vicinity of the interface, and after short period of time this region becomes completely depleted of OH^−^ ions and more remote anions must be taken to maintain constant current flow. Due to the sluggish anion diffusion in electrolytes, remote anions require higher energy (higher overpotential), and this leads to the enlarging of the micro-discharges. When micro-discharges reach the outer layer, forming big arcs, even slow [SiO_2_(OH)_2_]^2−^ anions are injected to discharge channels. A similar situation is observed when PEO is run in DC conditions. This may explain the smaller size of the soft-type micro-discharges compared to the regular ones and the high silicon content in oxide coatings obtained in DC PEO. Therefore, production of OH^−^ ions in the vicinity of the barrier layer by cathodic polarization has a quenching effect preventing formation of big arcs during PEO.

Since, as previous experiments showed, the stability of SSS is mainly governed by the anodic-to-cathodic charge ratio, it can be concluded from the above that in order to maintain SSS, a sufficient amount of OH^−^ accumulated in the oxide coating available for anodic reactions is required. Reaction (2) produces 1 mole of OH^−^ per 1 mole of electrons, while reaction (3) consumes 1 mole of OH^−^ per 1 mole of electrons. Assuming that reactions (2) and (3) are the only reactions that take place during the cathodic and anodic cycle, respectively, an equal charge for the cathodic and anodic reactions (R_C/A_ = 1) should satisfy the maintenance of SSS. Based on experiments, the minimum value of R_C/A_ to maintain SSS is 1.11; therefore, both values are quite convergent. In fact, besides reactions (2) and (3), there are other reactions, which are indicated by low Faradaic efficiency of oxide formation, which may be the source of this discrepancy.

**Figure 10:**
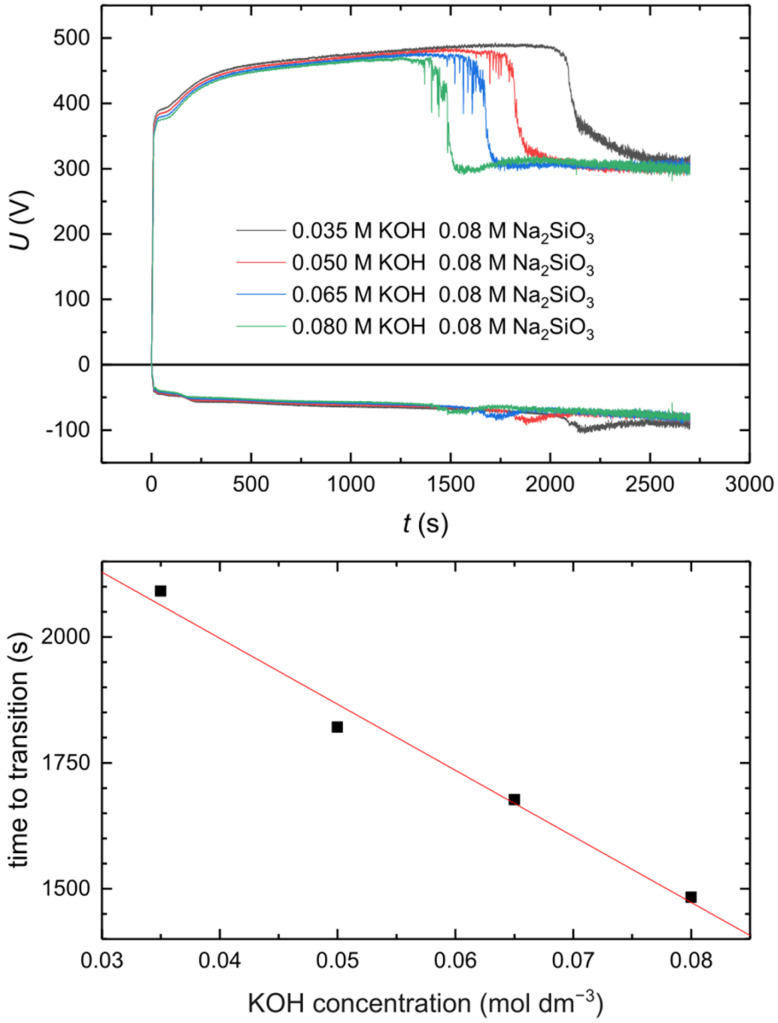
Effect of the electrolyte alkalinity on the time to SSS transition. Process parameters: j_A av_ = 10 A cm^−2^, f = 100 Hz, R_C/A_ = 1.25, D_A_, D_C_ = 50%.

In Figure 10, cell voltages during AC PEO performed at different concentrations of potassium hydroxide in the electrolytes, with the concentration of sodium silicate kept at the same level of 0.08 M, are presented. The higher the alkalinity of the electrolytes, the shorter the time required for transition to SSS, and it is visible that this relationship is quite linear. Monfort et al. [25] reported that higher concentrations of KOH in electrolytes provides a lower growth rate of the oxide layer. Taking this into account, as well as the fact that SSS requires a certain minimal volume of the oxide layer (volume of voids for OH^−^ ion accumulation), this relationship may be explained as follows. In fact, the real growth rate of the oxide layer in more alkaline electrolytes is higher, but due to the higher chemical dissolution rate of oxide in such electrolytes, the apparent growth rate is lower. Therefore, in more alkaline electrolytes, a certain level of voids in the oxide layer may be achieved after a shorter period of time. Another interesting observation is that anodic voltage during SSS does not depend on the electrolyte alkalinity but rather on R_C/A_, as was previously shown [20]. Therefore, the concentration of OH^−^ ions in the vicinity of the interface is determined by the cathodic-to-anodic reaction charge ratio, not by the concentration of OH^−^ ions in the bulk electrolytes, in the investigated range of the KOH concentration.

Another very important aspect, also raised in previous works, is the transportation of substrates through the oxide layer. Since water is a source of oxygen for oxide formation, its molecules must be delivered to the interface where charge transfer may occur. This transport is possible due to the relatively high liquid permeability of the oxide coating, not only of the outer porous part but also the intermediate dense part, as reported by Matykina et al. [26,27]. As the diffusion of water molecules through the oxide layer is sluggish, it may be responsible for the stochastic movement of micro-discharges during SSS. The transport rate of fresh electrolytes toward the metal/oxide interface is lower than the rate of reactions that lead to the depletion of water; thus, active sites move from depleted regions to refreshed regions.

To obtain a better overview of the reactions taking place during the PEO process, it would be necessary to look at the analysis of gaseous reaction products. Snizhko et al. [28,29] analyzed gaseous products during PEO, showing that they predominantly consisted of oxygen (93–95 at.%), with hydrogen content not exceeding 2–3 at.%. The PEO process in these works was performed with DC using diluted KOH electrolytes without sodium silicate addition. In another paper by Snizhko [30], the author used DC in pulsed and AC mode, and KOH-, Na_2_P_2_O_7_-, Na_2_SiO_3_-based electrolytes; hence, the conditions were more similar to those presented in this paper. The gaseous product composition predominantly consisted of hydrogen (~90%), even under DC conditions. The author ascribed the origin of hydrogen mainly to thermochemical reactions such as water decomposition and aluminum conversion. During gas analysis experiments, SSS was not reached. Our observations indicate lower gas evolution on the electrode during SSS than before; however, proper gas analysis experiments must be performed in order to present specific data about changes in the volume and composition of gases after transition.

### 3.4. Micro-Discharges During SSS

There are several indications that allow us to suppose that soft-type micro-discharges may have a different origin and nature than regular micro-discharges:

1. The optical emission during SSS has a continuous spectrum compared to the sharp peaks visible in the emission spectra of regular micro-discharges.
2. The anodic voltage during SSS is lower than the first spark voltage at the beginning of the process, when the first micro-discharge events occur.
3. No or much lower acoustic emission is audible during the soft-sparking state.

In case of the first point, some authors [31,32,33] pointed out that soft micro-discharges resemble the galvanoluminescence phenomenon, which also produces a continuous spectrum of light emission. Considering the significant amount of heat generated locally in active sites, visible micro-discharges can be in fact heated oxide, which generates an optical emission due to the thermoluminescence of alumina [34,35], which is related to galvanoluminescence. Another explanation for the disappearance of sharp peaks in the spectra after the transition to SSS is a screening effect of the outer parts of the oxide layer, since micro-discharges occur in deeper parts of the coating.

In the case of the second point, it is clearly visible from Figure 1 that the anodic voltage during SSS is around 300 V, whereas the voltage at which the first sparks appear is significantly higher—380–390 V. This indeed could suggest different mechanisms of discharge formation; however, the explanation can be simpler. Monfort et al. [25] showed that the higher the concentration of KOH in electrolytes (with the same concentration of Na_2_SiO_3_) the lower the first spark voltage and breakdown voltage. The concentration of KOH increasing from 0 to 0.15 M caused a decrease in the first spark voltage from 420 to 290 V. Monfort et al. did not explain the source of breakdown voltage decrease, but it may be explained by the formation of thinner barrier layer due to the higher dissolving properties of the electrolytes. Assuming a higher concentration of OH^−^ ions in the vicinity of the barrier layer than in the bulk electrolytes, the same effect may be observed in SSS; therefore, the thinning effect of the barrier layer by OH^−^ ions during SSS could explain the drop in anodic voltage.

In the case of the third point, a smaller size of soft-type micro-discharges cannot explain a lower acoustic emission, since even small micro-discharges (regular type) at the beginning of PEO generate high acoustic emission. Since gases are far better acoustic pressure carriers than liquids, the acoustic wave is mainly transferred by evolving gas bubbles and not by electrolytes. Taking this into account, it may be assumed that soft-type micro-discharges are not accompanied by gas evolution; therefore, the gas observed during SSS is generated during cathodic polarization.

### 3.5. Mechanism of Soft Sparking

**Figure 11:**
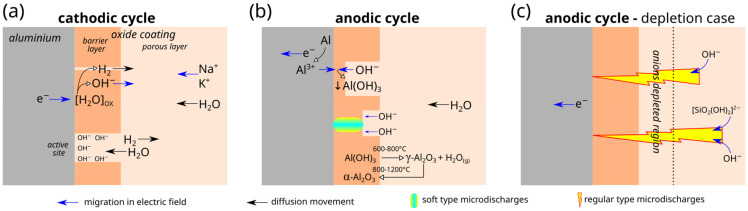
Concept scheme of ion transport during oxide-layer formation in AC mode: (**a**) cathodic cycle; (**b**) anodic cycle; (**c**) anodic cycle—depletion case.

Based on the experimental findings, the following mechanism of the AC PEO, including the soft-sparking state, schematically depicted in Figure 11, is suggested. During the cathodic cycle, deprotonation of water molecules occurs based on reaction (2) at the surface of the barrier layer (Figure 11a). In this process, active sites are being created in the barrier layer by evolving hydrogen, where the water diffusion toward the interface is later facilitated. Regions of active sites are more highly alkalized due to the accumulation of OH^−^ ions. In the subsequent anodic cycle, the hydroxide ions located in the vicinity are oxidized based on reaction (3) (Figure 11b). Deposited aluminum hydroxide seals active sites and suppresses repetitive breakdowns at these places, leading to the randomization of micro-discharge sites.

Due to the exothermic character of the oxidation reaction and Joule heat as an effect of the local passage of large current through the highly resistive barrier layer, a substantial amount of heat is generated at the interface. Due to the relatively high thermal resistance of the outer porous part of the coating, the removal of this heat to electrolytes is impeded. The presence of high temperature in this region enhances the further dehydration of Al(OH)_3_ to stable forms of alumina, mainly α- and γ-Al_2_O_3_.

An increased concentration of OH^−^ ions at the interface may lead to a decrease in voltage during anodic polarization due to (i) OH^−^ ions in the vicinity of the barrier layer requiring less energy to be discharged than anions placed at more remote positions, and (ii) the barrier layer undergoing local thinning by chemical dissolution additionally assisted by heat. These two effects may explain the anodic voltage drop after switching to a soft-sparking state.

In the case of an insufficient supply of OH^−^ ions in the vicinity of the barrier layer during the cathodic cycle (e.g., a too-low R_C/A_), in the course of the subsequent anodic polarization, more remote anions must be taken to maintain the charge flow, which leads to the expansion of the micro-discharges to larger arcs accompanied by an increase in anodic voltage during PEO (Figure 11c). Therefore, to maintain SSS, the vicinity of the barrier layer cannot be depleted too much of OH^−^ ions.

## 4. Conclusions

The explanation of the soft-sparking phenomenon in AC PEO, based on experimental observations of the soft-sparking state (SSS) behavior under different process conditions, is outlined above. This leads to a proposal regarding the role of cathodic current during oxide-layer formation. The main conclusions can be summarized as follows:

Stability of the soft-sparking state (SSS)

- The stability of SSS is primarily governed by the ratio of anodic to cathodic reactions in AC cycles, which influences substrate and product accumulation within oxide layer pores.

Role of cathodic polarization

- The electrochemical reduction of water at the metal/oxide interface generates hydrogen, inducing mechanical stress and creating defects in the barrier layer.
- These defects act as active sites with lower ohmic resistance, facilitating water transport and enhancing oxidation reactions in subsequent anodic polarization.
- Deprotonated water molecules serve as a better oxygen source during anodic reactions, potentially explaining the increased oxide layer growth rate during SSS.

Anodic polarization and micro-discharge behavior

- Anodic polarization seals active sites via Al(OH)_3_ deposition, leading to the randomization of micro-discharge locations. This cycle of active site formation and suppression occurs over several AC cycles.
- Soft and regular micro-discharges coexist before transitioning to SSS, after which only soft-type discharges persist.
- The transition to SSS occurs when the oxide layer accumulates enough OH^−^ ions during cathodic polarization to prevent reliance on more distant anions (e.g., [SiO_2_(OH)_2_]^2^^−^) in anodic reactions. This maintains SSS and lowers anodic voltage.
- Higher OH^−^ concentrations near the interface suppress soft micro-discharges, preventing their growth into larger arcs.

Structural transformations in the oxide layer

- The dehydration of the oxide layer begins during cathodic polarization and continues due to high temperatures at the metal/oxide interface, leading to stable alumina formation.
- Stochastic movement of soft-type micro-discharges during SSS may result from sluggish electrolyte diffusion through the oxide layer due to limited permeability.

Influence of process parameters

- Shorter cathodic pulse durations increase active site formation, leading to more dispersed micro-discharges.
- Reduced acoustic emission during SSS suggests minimal gas evolution, as gas bubbles facilitate acoustic wave propagation.

## Data Availability

The original contributions presented in this study are included in the article. Further inquiries can be directed to the corresponding author.

## Abbreviations

The following abbreviations are used in this manuscript:

PEO	plasma electrolytic oxidation
DC	direct current
AC	alternate current
SSS	soft sparking state
tss	time to transition
EIS	electrochemical impedance spectroscopy
